# Urban Movement and Alcohol Intake Strongly Predict Defaulting from Tuberculosis Treatment: An Operational Study

**DOI:** 10.1371/journal.pone.0035908

**Published:** 2012-05-02

**Authors:** Ibrahim Sendagire, Maarten Schim Van der Loeff, Andrew Kambugu, Joseph Konde-Lule, Frank Cobelens

**Affiliations:** 1 Directorate of Health, Kampala Capital City Authority, Kampala, Uganda; 2 Department of Global Health, Academic Medical Center, Amsterdam, The Netherlands; 3 Center for Infection and Immunity Amsterdam, Academic Medical Center, Amsterdam, The Netherlands; 4 Department of Research, Cluster Infectious Diseases, Public Health Service of Amsterdam, Amsterdam, The Netherlands; 5 Infectious Diseases Institute, School of Medicine, Makerere University College of Health Sciences, Kampala, Uganda; 6 School of Public Health, Makerere University College of Health Sciences, Kampala, Uganda; 7 Amsterdam Institute for Global Health and Development, Amsterdam, The Netherlands; San Francisco General Hospital, University of California San Francisco, United States of America

## Abstract

**Background:**

High levels of defaulting from treatment challenge tuberculosis control in many African cities. We assessed defaulting from tuberculosis treatment in an African urban setting.

**Methods:**

An observational study among adult patients with smear-positive pulmonary tuberculosis receiving treatment at urban primary care clinics in Kampala, Uganda. Defaulting was defined as having missed two consecutive monthly clinic visits while not being reported to have died or continued treatment elsewhere. Defaulting patients were actively followed-up and interviewed. We assessed proportions of patients abandoning treatment with and without the information obtained through active follow-up and we examined associated factors through multivariable logistic regression.

**Results:**

Between April 2007 and April 2008, 270 adults aged ≥15 years were included; 54 patients (20%) were recorded as treatment defaulters. On active follow-up vital status was established of 28/54 (52%) patients. Of these, 19 (68%) had completely stopped treatment, one (4%) had died and eight (29%) had continued treatment elsewhere. Extrapolating this to all defaulters meant that 14% rather than 20% of all patients had truly abandoned treatment. Daily consumption of alcohol, recorded at the start of treatment, predicted defaulting (adjusted odds ratio [OR^adj^] 4.4, 95%CI 1.8–13.5), as did change of residence during treatment (OR^adj^ 8.7, 95%CI 1.8–41.5); 32% of patients abandoning treatment had changed residence.

**Conclusions:**

A high proportion of tuberculosis patients in primary care clinics in Kampala abandon treatment. Assessing change of residence during scheduled clinic appointments may serve as an early warning signal that the patient may default and needs adherence counseling.

## Introduction

Retaining patients on tuberculosis (TB) treatment is a major challenge in many countries. Interruption of treatment has been associated with failure and death [1]–[3]. Levels of defaulting of up to 26% have been reported in Africa [4]–[5]. Defaulting levels tend to be especially high in urban areas. In Uganda, an estimated 14% of the new smear-positive patients defaulted from treatment in 2006 [6], while in Kampala city, where a quarter of the notified TB cases in Uganda are registered, defaulting levels are around 20% [7].

Studies have shown TB to be a stigmatized disease, which complicates control efforts [8]–[9]. Improved patient counseling and communication, patient choice of treatment supporter, and reinforcement of supervision activities are associated with improved treatment outcomes [10]. Poor treatment outcomes for patients on TB treatment threaten tuberculosis control efforts. The World Health Organization (WHO) target of successfully treating 85% of diagnosed patients cannot be achieved if defaulting levels remain high [11].

Defaulting as reported by TB control programs is based on proportions of patients who missed two consecutive monthly clinic visits during their treatment. In reality some of these patients may have died, or continued treatment elsewhere. In order to fully appreciate the magnitude of defaulting one has to correct the reported defaulting levels for deaths and continuation of treatment elsewhere based on active follow-up of patients. Understanding the pattern of defaulting has the potential of improving treatment outcomes. We conducted an observational study among patients on TB treatment at the primary health care level in Kampala to assess the level of defaulting, the proportion of patients reported as defaulters that had indeed abandoned treatment, and the factors associated with defaulting.

## Methods

### Ethical review

This study received ethical approval from the Medical Ethics Committees of Makerere University School of Public Health and the Academic Medical Centre, Amsterdam, Netherlands. Written informed consent was obtained from all participants.

### Study setting

The study was conducted at three of ten health centres operated by Kampala City Council (KCC) offering free outpatient services [12]. Located in a semi-urban area (Kiruddu), in the middle of a densely populated low-income area (Kisenyi) and in an industrial area (Kiswa) of the city, respectively, these centres were purposefully selected to reflect the varying socio-demographic conditions of the city.

Diagnosis of pulmonary TB was by direct smear microscopy. A standard 8 months treatment regimen (2RHZE/6HE) was used as first line treatment [13]. Patients were routinely counseled about stopping alcohol before initiating TB treatment. Direct observation of therapy was done by a treatment supporter, generally a family member or friend of the patient. The patients' particulars including drug regimen, treatment supporter's name and mobile telephone contacts were recorded in the health unit TB register.

### Study design

We conducted a prospective observational study following up smear-positive TB patients during treatment. Consenting patients ≥15 years, with new sputum smear-positive pulmonary TB, resident in Kampala city or within a radius of 16 km from the city centre were enrolled consecutively from April 1, 2007 to April 15, 2008. Excluded from the study were patients attending clinics outside study days, patients with mental illness or history of tuberculosis treatment for ≥1 month, and those below 18 years for whom parents/guardians could not be reached to give additional consent.

Data were collected by trained interviewers using pretested questionnaires in semi-structured interviews within eight weeks of starting TB treatment and at 2 months, 5 months and 8 months into treatment coinciding with scheduled treatment visits. Defaulters were followed up by the study team through home visits and interviewed if found alive. Those found to be off treatment were encouraged to resume treatment. Data on patients who died or could not be traced, but were presumed to be alive, were collected from relatives, friends or neighbors. Data were collected on demographic, socio-economic and functional status, treatment support, and social habits. Participants' opinions of the health service were assessed using a visual analogue scale (VAS) in which the participants were asked to confirm their opinion on the shown scale marked from one to 100.

### Definitions

A defaulter was defined according to the National TB and Leprosy Program (NTLP) as a patient who missed two or more consecutive monthly visits. We defined a patient as having abandoned treatment as a defaulter for whom the follow-up interview showed that he/she had completely stopped treatment for at least two consecutive months [14].

### Statistical analysis

Data were double entered; statistical analyses were carried out using Stata v10 (StataCorp, College Station, TX, USA). Proportions of patients defaulting from treatment according to the NTLP definition were calculated. Subsequently, proportions were calculated according to two alternative scenarios: (1) assuming that all patients not found on follow-up had abandoned treatment and (2) assuming that the probabilities of having died or having continued treatment elsewhere among the patients who were not found, were identical to those observed among those found. Associations between defaulting and explanatory variables were assessed using univariable and multivariable logistic regression also based on two scenarios. In the first scenario we included as defaulters all patients known to have abandoned treatment plus those who defaulted but could not be found on follow-up. In the second scenario we included as defaulters only those patients known to have abandoned treatment. Variables were included in the final models if they contributed to the model likelihood at p<0.05, or confounded the associations between defaulting and any of the other variables that were retained in the model. Age and sex were included the final models irrespective of their association or confounding effects.

## Results

### Study population

**Figure 1:**
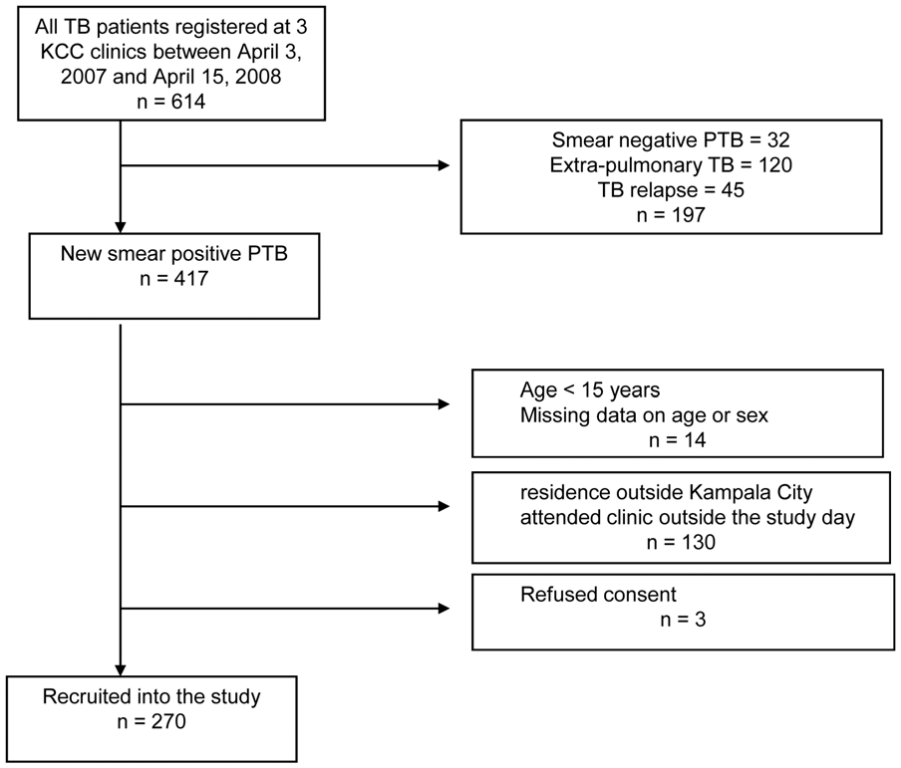
Flow chart showing the patients included in the defaulting to tuberculosis treatment study, Kampala, Uganda 2007–2008. KCC = Kampala City Council, TB = Tuberculosis, PTB = Pulmonary tuberculosis.

During the study period 614 patients were registered for TB treatment at the three clinics, of whom 417 (67.9%) had new smear-positive TB (figure 1). Fourteen patients were aged less than 15 years or had incomplete data on age and sex; complete data were available for 403 patients. One hundred thirty patients did not fit the inclusion criteria or attended clinic outside predefined study days, three patients refused consent and data were unavailable for 14 (Figure 1). Two hundred seventy patients (67.0% of those with complete data) were enrolled; table 1 shows their baseline characteristics. There were no significant differences in age (p = 0.359) and sex (p = 0.752) between the 270 patients included in the analysis and the 133 patients who were not. From the eligible patients a smaller proportion was recruited in Kiswa (58.3%), compared to Kisenyi (70.0%) and Kiruddu (76.7%; p = 0.016).

**Table 1:** Baseline characteristics of 403 smear-positive pulmonary tuberculosis patients registered for treatment at three Kampala City Clinics, Uganda.

	Included in analysis N (%)	Not included in analysis N (%)	p value
	270	133	
Median age	30(IQR 25–38)	32(IQR 24–48)	0.249
Age group			0.359
15–24 years	65 (24.1)	32 (25.0)	=
25–34 years	113 (41.9)	42 (32.8)	=
35–44 years	65 (24.1)	41 (32.0)	=
45–54 years	18 (6.7)	10 (7.8)	=
≥55 years	9 (3.3)	3 (2.4)	=
Sex			0.752
Male	160 (59.3)	81 (60.9)	=
Female	110 (40.7)	52 (39.1)	=
Education status			
None - primary 4	60 (22.2)	-	=
Primary 5–7	85 (31.5)	-	=
Senior 1–4	85 (31.5)	-	=
> senior 4	40 (14.8)	-	=
Marital status			
Single	94 (34.8)	-	=
Married/cohabiting	116 (43.0)	-	=
Divorced/Separated/widowed	60 (22.2)	-	=
Employment status			
Unemployed	91 (33.7)	-	=
Employed	179 (66.3)	-	=
WAB staging at initiation of TB treatment			
Working	252 (93.3)	-	=
Ambulatory	3 (1.1)	-	=
Not staged	15 (5.6)	-	=
Has treatment supporter			
No	34 (12.6)	-	=
Yes	236 (87.4)	-	=
Household wealth			
Lowest tertile	86 (34.0)	-	=
Middle tertile	85 (33.6)	-	=
Highest tertile	82 (32.4)	-	=
City clinic offering TB treatment			0.016
Kiruddu	46 (17.0)	14 (10.5)	=
Kisenyi	143 (53.0)	61 (45.9)	=
Kiswa	81 (30.0)	58 (43.6)	=

IQR = interquartile range.

TB = tuberculosis.

WAB = working, ambulatory, bedridden.

The median age of the 270 participants at baseline was 30 years (interquartile range [IQR] 25–38); 160 (59.3%) were male. Forty-six (17.0%) were recruited from Kiruddu, 143 (53.0%) from Kisenyi and 81 (30.0%) from Kiswa. The patients did not differ significantly between the three clinics in terms of sex (p = 0.861) and age (p = 0.938). The majority of patients, 236 (87.4%), reported having a treatment supporter.

Sixty-nine (25.6%) patients registered for treatment at the KCC clinics had been diagnosed at the main referral hospital for Kampala. The remaining 201 (74.4%) patients were both diagnosed and registered for treatment at a KCC clinic. These two groups of patients did not differ significantly by sex (p = 0.412) nor by KCC clinic where they registered (p = 0.440). The median age of patients diagnosed at the referral hospital was 28 years (IQR 25–33), which was lower than of those diagnosed at the city clinics, 32 years (IQR 25–39; p = 0.021).

### Defaulting

Of the 270 included patients, 54 (20.0%) defaulted from TB treatment according to the NTLP definition. On active follow-up, vital status was established for 28/54 (51.9%) patients. Of these, 19 (67.9%) had abandoned treatment, one (3.6%) had died and eight (28.6%) had continued treatment elsewhere. Of the 19 patients who had abandoned treatment one died after the planned end date of the continuation phase. In the first scenario (in which patients found to have abandoned treatment plus those not found were considered to be defaulters), the estimated proportion of defaulting in the whole cohort was 45/270 (16.7%). In the second scenario (in which patients found to have abandoned treatment plus similar proportions among those not found were considered to be defaulters), the estimated proportion of defaulting was 37/270 (13.7%, table 2).

**Table 2:** Defaulting among 270 smear-positive pulmonary TB patients, Kampala 2007–2008.

	Cure	Completed	Failure	Default	Death	Transfer out	Total
Defaulting as per program definition							
N	169	29	10	**54**	7	1	270
%	62.6	10.7	3.7	**20.0**	2.6	0.4	100
Alternative scenario 1: defaulters = patients not on treatment+patients not found							
N	169	29	10	**45**	8	9	270
%	62.6	10.7	3.7	**16.7**	3.0	3.3	100
Alternative scenario 2: defaulters = patients not on treatment+equivalent proportions among patients not found							
N	169	29	10	**37**	9	16	270
%	62.6	10.7	3.7	**13.7**	3.3	6.0	100

TB = tuberculosis.

### Factors associated with defaulting

In the analysis using the approach of scenario 1 to mark defaulters (not traced equals abandoning treatment), taking alcohol daily was significantly associated with defaulting from TB treatment in the univariable analysis (OR 4.4; 95%CI 1.9–10.2). Female sex was associated with a decreased likelihood of defaulting (OR 0.5; 95%CI 0.2–1.0). No other socio-demographic or program factors were significantly associated with defaulting in univariable analysis (table 3). In the multivariable analysis for this scenario drinking alcohol daily remained significantly associated with defaulting (OR^adj^ 4.9; 95%CI 1.8–13.5) with a dose response pattern observed for the odds ratios of the two categories of alcohol consumption compared to taking no alcohol at all. None of the remaining socio-demographic and program factors was significantly associated with defaulting.

**Table 3:** Associations of defaulting with baseline characteristics of 270 patients included in the defaulters to TB treatment study, Kampala 2007–2008.

Characteristic	Defaulted		Univariable analysis		Multivariable analysis		
	Yes	No					
	N (%)	N (%)	unadjusted	95% CI	Adjusted	95% CI	p-value*
	45	225	Odds ratio		Odds ratio		
Age group							
16–29 years	21 (17.1)	102 (82.9)	1		1		0.434
30–39 years	17 (17.9)	78 (82.1)	1.1	0.5–2.1	0.7	0.3–1.7	-
≥40 years	7 (13.5)	45 (86.5)	0.8	0.2–1.9	0.5	0.2–1.5	-
Sex							
Male	33 (20.6)	127 (79.4)	1		1		0.143
Female	12 (10.9)	98 (89.1)	0.5	0.2–1.0	0.5	0.2–1.3	=
Marital status							
Married/cohabiting	17 (14.7)	99 (85.3)	1		1		0.735
Divorced/Separated	12 (20.0)	48 (80.0)	1.5	0.6–3.3	1.1	0.6–3.8	=
Single	16 (17.0)	78 (83.0)	1.2	0.6–2.5	1.5	0.4–2.8	=
Employment status							
Employed	15 (16.5)	76 (83.5)	1		1		0.826
Unemployed	30 (16.8)	149 (83.2)	1.0	0.5–2.0	0.9	0.4–2.1	=
Other ailments (N = 267)							
None	31 (14.6)	181 (85.4)	1				-
HIV infection	9 (24.3)	28 (75.7)	1.9	0.8–4.4	-	-	-
Others	5 (27.8)	13 (72.2)	2.2	0.7–6.7	-	-	-
Transport cost (N = 255)							
0–500 UgShs	17 (12.9)	115 (87.1)	1		1		0.632
501–1000 UgShs	14 (18.2)	63 (81.8)	1.5	0.7–3.3	1.4	0.6–3.2	=
>1000 UghShs	8 (17.4)	38 (82.6)	1.4	0.6–3.6	1.5	0.6–3.9	=
Family size							
1–4 members	33 (19.1)	140 (80.9)	1		-	-	-
>4 members	12 (12.4)	85 (87.6)	0.6	0.3–1.2	-	-	-
Drinking alcohol (n = 267)							
Never drinks	10 (8.9)	102 (91.1)	1		1		0.007
1–3 days/week	14 (15.7)	75 (84.3)	1.9	0.8–4.5	2.2	0.8–5.9	
Drinks daily	20 (30.3)	46 (69.7)	4.4	1.9–10.2	4.9	1.8–13.5	
Type of facility diagnosing TB							
City clinic	37 (18.4)	164 (81.6)	1		-	-	-
Hospital	8 (11.6)	61 (88.4)	0.6	0.3–1.3	-	-	-

* = p-value for variables included in the final model.

CI = confidence interval.

TB = tuberculosis.

UgShs = Ugandan Shillings.

In the additional analysis excluding the 26 patients that were not found during follow-up (leaving 243 patients, among them 19 defaulters), the patterns of associations with defaulting were similar. However the association with alcohol intake was weaker and no longer statistically significant (p = 0.459, Table S1). When we ran the multivariable logistic regression model on the 26 patients whom we failed to locate, we found similar adjusted odds ratios for most variables, but a stronger and significant association between drinking alcohol and defaulting (compared to no alcohol use: OR^adj^ 3.4 for drinking 1–3 days per week; OR^adj^ 9.1 for drinking daily [p = 0.004, Table S2]).

### Commonest reasons identified for defaulting

All patients reported having taken only fixed dose combinations of their medication. Of the 18 defaulters found and interviewed, 10 (56%) still had treatment supporters at the time they stopped taking their medication. No patients had made payments in order to access a clinician, drugs, medical refill or clinical review. The reasons for defaulting that patients mentioned most frequently were started feeling better (5 patients), side effects of TB drugs (3), work conflict (1) and abandoned treatment supporter (1). The median time the patient spent during the last refill of drugs was 17.5 minutes (IQR 15–30) while the median waiting time before being attended to was 20 minutes (IQR 15–30). Half of the defaulters had cough, fever or weight loss at the time of the interview after defaulting. The defaulters had a favorable opinion of the health services offered at the city clinics (table 4).

**Table 4:** History details of 18 defaulters from tuberculosis treatment by the time of the first interview after defaulting, Kampala, 2007–2008.

	N	%
Patient characteristic		
1. Had cough at the time of interview after defaulting	12	66.7
2. Had chest pain at the time of interview after defaulting	10	55.6
3. Had fever at the time of interview after defaulting	9	50.0
4. Loss of weight at the time of interview after defaulting	10	55.6
5. Had difficulty in breathing	5	27.8
6. Had changed residence since?	5	27.8
7. Marital status had changed since?	2	11.1
8. Employment status had changed since?	5	27.8
9. Stopped taking treatment during the intensive phase	6	33.3
10. Willingness to get back on treatment	11	61.1
11. Had a treatment supporter now or ever?	10	55.6

Patient opinion of the health worker (median, mean) using VAS	Median	Mean
1. Being listened to	90.0	83.6
2. Being given a chance to explain one's problems	90.0	82.0
3. Being treated with respect	90.0	85.3
4. Trust in the health worker	89.0	85.0
5. Being given adequate privacy during consultations	90.0	84.2
6. Friendliness from the health care worker	90.0	82.6

Service delivery at the health centre (median, mean) using VAS	Median	Mean
1. Time spent in minutes during the last refill appointment	17.5	23.9
2. Waiting time in minutes before being attended to	20.0	20.6
3. Rating how good the service is at the clinic using the VAS	80.0	82.8

TB = tuberculosis.

VAS = visual analogue scale.

Of note 2 of 18 defaulters who were known to have abandoned treatment and 9 of 26 defaulters who could not be traced had changed residence (11/44, 25.0%) during treatment. At any point in time during treatment or at follow-up, change of residence was recorded for 42/270 patients (15.6%), including one of eight defaulters who had in fact continued treatment elsewhere (12.5%). Since this provided a potential entry for intervention we further explored the role of changing residence. Defaulting was strongly associated with change of residence during treatment in univariate analysis (OR 12.6; 95%CI 4.1–38.9) and after adjustment for age, transport costs, alcohol consumption, marital status, and employment status (OR 23.2; 95%CI 4.5–119.0, table 5). In an analysis examining change of residence established during scheduled treatment visits only (i.e. excluding follow-up visits that were made purely for research purposes), change of residence was still significantly associated with defaulting (crude OR 4.8 (95%CI 1.4–16.6) and adjusted OR 8.7 (95%CI 1.8–41.5)).

**Table 5:** Associations between change of residence and defaulting from tuberculosis treatment among patients included in the defaulters to TB treatment study, Kampala 2007–2008.

Characteristic	Defaulting (%)	Crude odds ratio (95% CI)	Adjusted odds ratio* (95% CI)
Change of residence ascertained during treatment visits or follow-up (N = 225)			
No	5/183 (2.7)	1	1
Yes	11/42 (26.2)	12.6 (4.1–38.9)	23.2 (4.5–119.0)
Change of residence ascertained during treatment visits only (N = 220)†			
No	6/184 (3.3)	1	1
Yes	5/36 (13.9)	4.8 (1.4–16.6)	8.7 (1.8–41.5)

* = adjusted for marital status, employment status and transport costs.

† = change of residence ascertained at the end of two months interval and two-five months interval.

CI = confidence interval.

TB = tuberculosis.

Of the 16 defaulters who changed residence, one was known to have continued treatment elsewhere. Since we only had this information for 8 patients whom we were able to interview, the true proportion of defaulters changing residence who continued treatment elsewhere was between 6.3% (1/16) and 56.2% (9/16).

## Discussion

One fifth of patients registered for treatment at three city clinics defaulted on TB treatment. This level of defaulting is within range of those reported by studies carried out in sub-Saharan Africa or other high TB incidence countries [4], [5], [10], [15]–[20]. Most of these reported defaulting levels are based on TB program definitions and not on follow-up of patients. When we made efforts to trace the defaulters, we found that 4% had died, and 29% had in fact continued treatment elsewhere. Thus, high defaulting levels in TB programs may mask unnoticed deaths but also treatment transfers to other clinics. Nonetheless, the finding that between 14% and 17% of patients abandoned treatment remains a concern.

Change of residence during treatment was strongly associated with defaulting as per program definition. At least 30% (16/54) of defaulters had changed residence, and of the 8 patients for whom this could be verified only one had continued treatment elsewhere. Thus, in an urban area such as Kampala, TB patients apparently often change residence, and this hampers continuation of treatment, e.g. due to lack of access to, or knowledge of, treatment options elsewhere. It is also possible that some patients in our study started treatment giving false addresses while in fact living outside the 16 km perimeter, and returned home when feeling better. On the other hand some patients could have returned to the village to die if they felt worse.

Nearly two thirds of the defaulters consumed alcohol, and 41% consumed alcohol daily. Daily alcohol consumption noted at the start of treatment was a strong predictor of defaulting (with frequency of alcohol consumption showing a dose-dependent relationship) while socio-demographic factors ascertained at the start of treatment were not. Some other studies also found that alcohol consumption was associated with defaulting [17]–[18], [21]. In our additional analyses it was having been lost to follow-up that showed a strong association with alcohol use rather than having been found and known to have abandoned treatment. This suggests that those who drink alcohol have a high risk of moving around or outside the city, of providing false addresses or of living in slum areas where addresses are difficult to locate. This may lead these patients to abandon treatment, although we cannot exclude that it also leads to increased mortality.

Treatment-related factors, some of qualitative nature, did not seem to explain why patients defaulted. The median waiting time for treatment visits of 20 minutes was surprisingly short in view of the crowded health centers in Kampala. Also in a study carried out in a Ghanaian hospital, waiting times were not associated with defaulting from TB treatment [16]. Half of the defaulters reported still having the same treatment supporter at the time they stopped taking their medication. No patient made payments before accessing a clinician or drugs. The majority had a favorable opinion about the clinic services. Matsubayashi et al. made similar observations in the same setting [22].

The associations between defaulting and alcohol consumption and between defaulting and change of residence, as well as the lack of other significant associations, suggest that defaulting has more to do with individual patient characteristics and practicalities of living in this urban environment than with health system barriers. A study from a similar setting in South Africa recommended a more accurate registration of addresses of patients at admission [23]. Since we found that defaulting was likely if a patient reported having changed residence during treatment, a potential area for intervention would be to routinely ask about (planned) change of residence during scheduled treatment appointments and informing patients in detail about their options of continuing treatment elsewhere, and introducing a follow-up system to check if patients transferred in at the other clinic.

Our study had limitations. A sizeable proportion of defaulters were not found during follow-up, potentially limiting the generalizability of our results. This was to some extent inevitable. We could have put into place a tracking system with higher likelihood of finding patients (e.g. by visiting their homes at start of treatment, weekly contact, or contacting multiple persons in the patient's social network) but by that we would have intervened in the routine process of TB treatment and probably have changed the defaulting patterns that were the subject of our study. TB patients were included at government health facilities only, where services are free. The study's findings cannot be generalized to settings where user fees apply, like private health facilities. Furthermore, the picture could possibly be different if other forms of TB and private health facilities were included. As the study included only smear-positive pulmonary patients, findings may not be extrapolated to smear-negative pulmonary TB patients or patients with extrapulmonary TB. Many of the deaths were not verified by a death certificate or post mortem report. The study area was limited to a radius of 16 km from the city centre, very typical of an urban area. This could possibly explain the lack of association between variables such as distance to health facility and transport costs with defaulting to TB treatment, which has been observed in referral hospital-based studies that include a mixture of rural and urban populations. It may well be that predictors of defaulting in urban populations are different from those in rural populations. Factors such as distance have been shown to be relevant for defaulting in studies done in rural populations [4], [24]. Our study findings can only be generalized to public primary health care facilities in urban areas.

Although ours was an observational study with no intervention, having been informed about the possibility of being actively followed (during the process of obtaining informed consent) may have made patients change their behavior. Prior to commencement of the study the sites had been improved through provision of adequate regular laboratory supplies for TB diagnostics. Additional clinic space was provided through containers set up by the study to improve patient privacy and confidentiality. These perceived improvements in health care could have influenced the patients favorably towards good adherence and completion of treatment. Finally, data on whether the patients moved outside the service area of the clinics or not were not available. However, change of residence is the reason why the majority of the patients could not be found.

We conclude that 20% of TB patients in three primary care clinics in Kampala missed two or more monthly clinic appointments. Even after excluding patients who in fact had died or continued treatment elsewhere, at least a sizeable 14% of patients abandoned treatment completely. Daily consumption of alcohol and change of residence predicted defaulting from TB treatment, in particular complete loss to follow-up for the TB program. Assessing change of residence during clinic appointments may serve as an early warning signal to care providers to increase support and direct resources towards linking patients to other clinics near their new residence.

## Supporting Information

Table S1 Associations of defaulting with baseline characteristics of 243 patients included in the defaulters to TB treatment study, Kampala 2007–2009**. ** = this analysis excludes the one death and 26 patients who were lost to follow up. CI = confidence interval. TB = tuberculosis. Ugshs = Ugandan Shillings.
(DOC)

Table S2 Associations of defaulting with baseline characteristics of 250 patients included in the defaulters to TB treatment study, Kampala 2007–2009***. *** = this analysis excludes the one death plus the 19 patients known to be defaulters with certainty. CI = confidence interval. TB = tuberculosis. Ugshs = Ugandan Shillings.
(DOC)
