# Supplementary material for: Urban Movement and Alcohol Intake Strongly Predict Defaulting from Tuberculosis Treatment: An Operational Study
Source: PLoS One. 2012 May 2;7(5):e35908. doi: 10.1371/journal.pone.0035908 (PMC3342307; doi:10.1371/journal.pone.0035908)
Supplement: Table S1 — Associations of defaulting with baseline characteristics of 243 patients included in the defaulters to TB treatment study, Kampala 2007–2009**. ** = this analysis excludes the one death and 26 patients who were lost to follow up. CI = confidence interval. TB = tuberculosis. Ugshs = Ugandan Shillings. (DOC) [file pone.0035908.s001.doc]

| **Table S1. Associations of defaulting with baseline characteristics of 243 patients included in the defaulters to TB treatment study, Kampala 2007-2009**** | | | | | | | | | | | | | | | | | | | | | | | | | | | | | | | | | | | | | | | | | | | | | | | | | | | | |  | |
| --- | --- | --- | --- | --- | --- | --- | --- | --- | --- | --- | --- | --- | --- | --- | --- | --- | --- | --- | --- | --- | --- | --- | --- | --- | --- | --- | --- | --- | --- | --- | --- | --- | --- | --- | --- | --- | --- | --- | --- | --- | --- | --- | --- | --- | --- | --- | --- | --- | --- | --- | --- | --- | --- | --- |
|  | | | | | | |  | | | | | | | | |  | | | | | |  | | | | | |  | | | | | | |  | | | | | |  | | | | | |  | | | | | |  | |
| **Characteristic** | | | | | | |  | | | **Defaulted** | | | | | | | | | | **Univariable analysis** | | | | | | | | | | **Multivariable analysis** | | | | | | | | | | | | | | | | | | |  | | | | |  |
|  | | | | | | |  | | **Yes** | | | | **No** | | | | | |  | | | | | |  | | | | | | |  | | | | | |  | | | | | |  | | | | | |  | | | | |
|  | | | | | | |  | | **N (%)** | | | | **N (%)** | | | | | | **Unadjusted** | | | | | | **95% CI** | | | | | | | **Adjusted** | | | | | | **95% CI** | | | | | | **p-value** | | | | | | | | |  | |
|  | | | | | | |  | | **19** | | | | **224** | | | | | | **Odds ratio** | | | | | |  | | | | | | | **Odds ratio** | | | | | |  | | | | | |  | | | | | |  | | | | |
|  | | | | | | |  | | | | | | | | |  | | | | | |  | | | | | |  | | | | | | |  | | | | | |  | | | | | |  | | | | | |  | |
| Age group | | | | | | | | | | | | | | | |  | | | | | |  | | | | | |  | | | | | | |  | | | | | |  | | | | | |  | | | | | |  | |
|  | 16 - 29 years | | | | | | | | 9 (8.2) | | | | 101 (91.8) | | | | | | 1 | | | | | |  | | | | | | | 1 | | | | | |  | | | | | | 0.400 | | | | | |  | | | | |
|  | 30 - 39 years | | | | | | | | 8 (9.3) | | | | 78 (90.7) | | | | | | 1.2 | | | | | | 0.4 - 3.1 | | | | | | | 0.8 | | | | | | 0.2 - 3.0 | | | | | | - | | | | | |  | | | | |
|  | ≥ 40 years | | | | | | | | 2 (4.3) | | | | 45 (95.7) | | | | | | 0.5 | | | | | | 0.1 – 2.4 | | | | | | | 0.3 | | | | | | 0.0 - 2.1 | | | | | | - | | | | | |  | | | | |
|  |  | | | | | | | | | |  | | | | | |  | | | | | |  | | | | | |  | | | | | | |  | | | | | |  | | | | | | |  | | | | |  |
| Sex | | | | | | | | | | | | | | | |  | | | | | |  | | | | | |  | | | | | | |  | | | | | |  | | | | | |  | | | | | |  | |
|  | | Male | | | | | | | 15 (10.6) | | | | 126 (89.4) | | | | | | 1 | | | | | |  | | | | | | | 1 | | | | | |  | | | | | | 0.104 | | | | | |  | | | | |
|  | | Female | | | | | | | 4 (3.9) | | | | 98 (96.1) | | | | | | 0.3 | | | | | | 0.1 - 1.1 | | | | | | | 0.3 | | | | | | 0.1 - 1.4 | | | | | | = | | | | | |  | | | | |
|  | | | | | | |  | | | | | | | | |  | | | | | |  | | | | | |  | | | | | | |  | | | | | |  | | | | | |  | | | | | |  | |
| Marital status | | | | | | | | | | | | | | | |  | | | | | |  | | | | | |  | | | | | | |  | | | | | |  | | | | | |  | | | | | |  | |
|  | | | Married/cohabiting | | | | | | | 8 (7.6) | | | | 98 (92.5) | | | | | | 1 | | | | | |  | | | | | | | 1 | | | | | |  | | | | | | 0.816 | | | | | | |  | | |
|  | | | Divorced/Separated | | | | | | | 5 (9.4) | | | | 48 (90.6) | | | | | | 1.3 | | | | | | 0.4 - 4.1 | | | | | | | 1.3 | | | | | | 0.3 – 5.3 | | | | | | = | | | | | | |  | | |
|  | | | Single | | | | | | | 6 (7.1) | | | | 78 (92.9) | | | | | | 0.9 | | | | | | 0.3 - 2.8 | | | | | | | 0.8 | | | | | | 0.2 – 3.0 | | | | | | = | | | | | | |  | | |
|  | | | | | | |  | | | | | | | | |  | | | | | |  | | | | | |  | | | | | | |  | | | | | |  | | | | | |  | | | | | |  | |
| Employment status | | | | | | | | | | | | | | | |  | | | | | |  | | | | | |  | | | | | | |  | | | | | |  | | | | | |  | | | | | |  | |
|  | | | | Employed | | | | | 4 (5.0) | | | | 76 (95.0) | | | | | | 1 | | | | | |  | | | | | | | 1 | | | | | |  | | | | | | 0.984 | | | | | |  | | | | |
|  | | | | Unemployed | | | | | 15 (9.2) | | | | 148 (90.8) | | | | | | 1.9 | | | | | | 0.6 - 6.0 | | | | | | | 1.0 | | | | | | 0.3 – 3.6 | | | | | | = | | | | | |  | | | | |
|  | | | | | | |  | | | | | | | | |  | | | | | |  | | | | | |  | | | | | | |  | | | | | |  | | | | | |  | | | | | |  | |
| Transport cost (N=255) | | | | | | | | | | | | | | | |  | | | | | |  | | | | | |  | | | | | | |  | | | | | |  | | | | | |  | | | | | |  | |
|  | | | | | 0 - 500 Ugshs | | | | 6 (5.0) | | | | 114 (95.0) | | | | | | 1 | | | | | |  | | | | | | | 1 | | | | | |  | | | | | | 0.318 | | | | | | |  | | | |
|  | | | | | 501-1000 Ugshs | | | | 9 (12.5) | | | | 63 (87.5) | | | | | | 2.7 | | | | | | 0.9 - 8.0 | | | | | | | 2.3 | | | | | | 0.7 - 7.2 | | | | | | = | | | | | | |  | | | |
|  | | | | | > 1000 Ugshs | | | | 2 (5.0) | | | | 38 (95.0) | | | | | | 1.0 | | | | | | 0.2 – 5.2 | | | | | | | 1.1 | | | | | | 0.2 - 5.8 | | | | | | = | | | | | | |  | | | |
|  | | | | | | |  | | | | | | | |  | | | | | |  | | | | | |  | | | | | | |  | | | | | |  | | | | | |  | | | | | | |  | |
| Drinking alcohol (n=267) | | | | | | | | | | | | | | | |  | | | | | |  | | | | | |  | | | | | | |  | | | | | |  | | | | | |  | | | | | |  | |
|  | | | | | | Never drinks | | 6 (5.6) | | | | 102 (94.4) | | | | | | 1 | | | | | |  | | | | | | | 1 | | | | | |  | | | | | | 0.459 | | | | |  | | | | | | |
|  | | | | | | 1-3 days/week | | 5 (6.3) | | | | 74 (93.7) | | | | | | 1.1 | | | | | | 0.3 – 3.9 | | | | | | | 1.4 | | | | | | 0.4 - 5.2 | | | | | |  | | | | |  | | | | | | |
|  | | | | | | Drinks daily | | 7 (13.2) | | | | 46 (86.8) | | | | | | 2.6 | | | | | | 0.8 – 8.1 | | | | | | | 2.5 | | | | | | 0.6 – 10.3 | | | | | |  | | | | |  | | | | | | |
|  | | | | | | |  | | | | | | | | |  | | | | | |  | | | | | |  | | | | | | |  | | | | | |  | | | | | |  | | | | | |  | |
