# Supplementary material for: Urban Movement and Alcohol Intake Strongly Predict Defaulting from Tuberculosis Treatment: An Operational Study
Source: PLoS One. 2012 May 2;7(5):e35908. doi: 10.1371/journal.pone.0035908 (PMC3342307; doi:10.1371/journal.pone.0035908)
Supplement: Table S2 — Associations of defaulting with baseline characteristics of 250 patients included in the defaulters to TB treatment study, Kampala 2007–2009***. *** = this analysis excludes the one death plus the 19 patients known to be defaulters with certainty. CI = confidence interval. TB = tuberculosis. Ugshs = Ugandan Shillings. (DOC) [file pone.0035908.s002.doc]

| **Table S2. Associations of defaulting with baseline characteristics of 250 patients included in the defaulters to TB treatment study, Kampala 2007-2009***** | | | | | | | | | | | | | | | | | | | | | | | | | | | | | | | | | | | | | | | | | | | | | | | | | | | | |  | |
| --- | --- | --- | --- | --- | --- | --- | --- | --- | --- | --- | --- | --- | --- | --- | --- | --- | --- | --- | --- | --- | --- | --- | --- | --- | --- | --- | --- | --- | --- | --- | --- | --- | --- | --- | --- | --- | --- | --- | --- | --- | --- | --- | --- | --- | --- | --- | --- | --- | --- | --- | --- | --- | --- | --- |
|  | | | | | | |  | | | | | | | | |  | | | | | |  | | | | | |  | | | | | | |  | | | | | |  | | | | | |  | | | | | |  | |
| **Characteristic** | | | | | | |  | | | **Defaulted** | | | | | | | | | | **Univariable analysis** | | | | | | | | | | **Multivariable analysis** | | | | | | | | | | | | | | | | | | |  | | | | |  |
|  | | | | | | |  | | **Yes** | | | | **No** | | | | | |  | | | | | |  | | | | | | |  | | | | | |  | | | | | |  | | | | | |  | | | | |
|  | | | | | | |  | | **N (%)** | | | | **N (%)** | | | | | | **unadjusted** | | | | | | **95% CI** | | | | | | | **Adjusted** | | | | | | **95% CI** | | | | | | **p-value** | | | | | | | | |  | |
|  | | | | | | |  | | **26** | | | | **224** | | | | | | **Odds ratio** | | | | | |  | | | | | | | **Odds ratio** | | | | | |  | | | | | |  | | | | | |  | | | | |
|  | | | | | | |  | | | | | | | | |  | | | | | |  | | | | | |  | | | | | | |  | | | | | |  | | | | | |  | | | | | |  | |
| Age group | | | | | | | | | | | | | | | |  | | | | | |  | | | | | |  | | | | | | |  | | | | | |  | | | | | |  | | | | | |  | |
|  | 16 - 29 years | | | | | | | | 13 (10.6) | | | | 110 (89.4) | | | | | | 1 | | | | | |  | | | | | | | 1 | | | | | |  | | | | | | 0.228 | | | | | |  | | | | |
|  | 30 - 39 years | | | | | | | | 9 (9.5) | | | | 86 (90.5) | | | | | | 0.9 | | | | | | 0.4 - 2.2 | | | | | | | 0.4 | | | | | | 0.1 – 1.4 | | | | | | - | | | | | |  | | | | |
|  | ≥ 40 years | | | | | | | | 4 (7.7) | | | | 48 (92.3) | | | | | | 0.7 | | | | | | 0.2 – 2.3 | | | | | | | 0.3 | | | | | | 0.1 – 1.4 | | | | | | - | | | | | |  | | | | |
|  |  | | | | | | | | | |  | | | | | |  | | | | | |  | | | | | |  | | | | | | |  | | | | | |  | | | | | | |  | | | | |  |
| Sex | | | | | | | | | | | | | | | |  | | | | | |  | | | | | |  | | | | | | |  | | | | | |  | | | | | |  | | | | | |  | |
|  | | Male | | | | | | | 19 (11.9) | | | | 141 (88.1) | | | | | | 1 | | | | | |  | | | | | | | 1 | | | | | |  | | | | | | 0.277 | | | | | |  | | | | |
|  | | Female | | | | | | | 7 (6.4) | | | | 103 (93.6) | | | | | | 0.5 | | | | | | 0.2 - 1.2 | | | | | | | 0.5 | | | | | | 0.2 - 1.7 | | | | | | = | | | | | |  | | | | |
|  | | | | | | |  | | | | | | | | |  | | | | | |  | | | | | |  | | | | | | |  | | | | | |  | | | | | |  | | | | | |  | |
| Marital status | | | | | | | | | | | | | | | |  | | | | | |  | | | | | |  | | | | | | |  | | | | | |  | | | | | |  | | | | | |  | |
|  | | | Married/cohabiting | | | | | | | 10 (8.6) | | | | 106 (91.4) | | | | | | 1 | | | | | |  | | | | | | | 1 | | | | | |  | | | | | | 0.792 | | | | | | |  | | |
|  | | | Divorced/Separated | | | | | | | 7 (11.7) | | | | 53 (88.3) | | | | | | 1.4 | | | | | | 0.5 – 3.9 | | | | | | | 1.5 | | | | | | 0.4 – 4.7 | | | | | | = | | | | | | |  | | |
|  | | | Single | | | | | | | 9 (9.6) | | | | 85 (90.4) | | | | | | 1.1 | | | | | | 0.4 - 2.9 | | | | | | | 0.9 | | | | | | 0.3 – 3.0 | | | | | | = | | | | | | |  | | |
|  | | | | | | |  | | | | | | | | |  | | | | | |  | | | | | |  | | | | | | |  | | | | | |  | | | | | |  | | | | | |  | |
| Employment status | | | | | | | | | | | | | | | |  | | | | | |  | | | | | |  | | | | | | |  | | | | | |  | | | | | |  | | | | | |  | |
|  | | | | Employed | | | | | 10 (11.0) | | | | 81 (89.0) | | | | | | 1 | | | | | |  | | | | | | | 1 | | | | | |  | | | | | | 0.938 | | | | | |  | | | | |
|  | | | | Unemployed | | | | | 16 (8.9) | | | | 163 (91.1) | | | | | | 0.8 | | | | | | 0.3 – 1.8 | | | | | | | 1.0 | | | | | | 0.3 – 2.8 | | | | | | = | | | | | |  | | | | |
|  | | | | | | |  | | | | | | | | |  | | | | | |  | | | | | |  | | | | | | |  | | | | | |  | | | | | |  | | | | | |  | |
| Transport cost (N=255) | | | | | | | | | | | | | | | |  | | | | | |  | | | | | |  | | | | | | |  | | | | | |  | | | | | |  | | | | | |  | |
|  | | | | | 0 - 500 Ugshs | | | | 11 (8.3) | | | | 121 (91.7) | | | | | | 1 | | | | | |  | | | | | | | 1 | | | | | |  | | | | | | 0.549 | | | | | | |  | | | |
|  | | | | | 501-1000 Ugshs | | | | 5 (6.5) | | | | 72 (93.5) | | | | | | 0.8 | | | | | | 0.3 – 2.3 | | | | | | | 0.8 | | | | | | 0.2 – 2.4 | | | | | | = | | | | | | |  | | | |
|  | | | | | > 1000 Ughshs | | | | 6 (13.0) | | | | 40 (87.0) | | | | | | 1.7 | | | | | | 0.6 – 4.7 | | | | | | | 1.6 | | | | | | 0.5 – 4.9 | | | | | | = | | | | | | |  | | | |
|  | | | | | | |  | | | | | | | |  | | | | | |  | | | | | |  | | | | | | |  | | | | | |  | | | | | |  | | | | | | |  | |
| Drinking alcohol (n=267) | | | | | | | | | | | | | | | |  | | | | | |  | | | | | |  | | | | | | |  | | | | | |  | | | | | |  | | | | | |  | |
|  | | | | | | Never drinks | | 4 (3.6) | | | | 108 (96.4) | | | | | | 1 | | | | | |  | | | | | | | 1 | | | | | |  | | | | | | 0.004 | | | | |  | | | | | | |
|  | | | | | | 1-3 days/week | | 9 (10.1) | | | | 80 (89.9) | | | | | | 3.0 | | | | | | 0.9 – 10.2 | | | | | | | 3.4 | | | | | | 0.8 – 14.1 | | | | | |  | | | | |  | | | | | | |
|  | | | | | | Drinks daily | | 13 (19.7) | | | | 53 (80.3) | | | | | | 6.6 | | | | | | 2.1 – 21.3 | | | | | | | 9.1 | | | | | | 2.2 – 37.9 | | | | | |  | | | | |  | | | | | | |
|  | | | | | | |  | | | | | | | | |  | | | | | |  | | | | | |  | | | | | | |  | | | | | |  | | | | | |  | | | | | |  | |
